# The Patient’s Perspective on the Effects of Intranasal Esketamine in Treatment-Resistant Depression

**DOI:** 10.3390/brainsci13101494

**Published:** 2023-10-22

**Authors:** Maria Pepe, Giovanni Bartolucci, Ilaria Marcelli, Francesco Pesaresi, Andrea Brugnami, Romina Caso, Alessia Fischetti, Flavia Grisoni, Marianna Mazza, Giovanni Camardese, Marco Di Nicola, Gabriele Sani

**Affiliations:** 1 Department of Neuroscience, Section of Psychiatry, Università Cattolica del Sacro Cuore, Largo Francesco Vito 1, 00168 Rome, Italy; 2 Department of Psychiatry, Fondazione Policlinico Universitario Agostino Gemelli IRCCS, Largo Agostino Gemelli 8, 00168 Rome, Italy

**Keywords:** glutamatergic system, mood disorders, patient experience, personalized medicine, psychopharmacology

## Abstract

The effectiveness of the esketamine nasal spray (ESK-NS) for treatment-resistant depression (TRD) has been confirmed by real-world studies. Available evidence derived from clinician-rated assessments might differ from patients’ perceptions about the helpfulness of treatments. We aimed to verify the effect of ESK-NS from patients’ view in 25 TRD patients (56% males, 55.1 ± 10.9 years) treated with ESK-NS (mean dose: 78.4 ± 11.43 mg) for three months and evaluated at different time-points through clinician-rated and self-administered scales, assessing changes in depression, anhedonia, sleep, cognition, suicidality, and anxiety. We observed an overall early improvement that lasted over time (endpoint total score reduction in Montgomery-Åsberg Depression Rating Scale, *p* < 0.001, Beck Depression Inventory, *p* = 0.003). Patients reported a significant self-rated decrease in anhedonia at two months (Snaith–Hamilton Pleasure Scale, *p* = 0.04) and in suicide ideation at endpoint (BDI subitem 9, *p* = 0.039) vs. earlier improvements detected by clinicians (one-month reduction in MADRS subitem 8, *p* = 0.005, and subitem 10, *p* = 0.007). These findings confirm the effectiveness of a three-month treatment with ESK-NS in TRD patients, highlighting an overall overlapping response from patients’ and clinicians’ perspectives, although with some differential effects on specific symptoms at given time-points. Including patients’ viewpoints in routine assessments could inform clinical practice, ensuring a better characterization of clinical phenotypes to deliver personalized interventions.

## 1. Introduction

Treatment-resistant depression (TRD) is a significant public health concern because of its prevalence, with variable rates of 12–55% [1], and the socioeconomic impact [2]. Commonly defined as a depressive episode that fails to respond to at least two antidepressants from different pharmacological classes, adequately assumed for time, dosage, and adherence [3,4], TRD may occur in both unipolar and bipolar depression and is characterized by heterogeneous pathophysiological dysfunctions [5].

Integrating the monoaminergic hypothesis of mood disorders, the role of the glutamatergic system has gained increasing attention in relation to TRD, and the efficacy of compounds antagonizing N-methyl-D-aspartate (NMDA) receptors has been reported [6]. Esketamine, the S-enantiomer of ketamine that acts as a noncompetitive NMDA-Rs antagonist with great affinity [7], has been found to improve TRD when administered with oral antidepressants in both randomized trials and real-world studies [2,8]. The intranasal formulation of Esketamine (ESK-NS) has been approved by several medical agencies as a therapeutic tool for TRD, and its favorable safety and tolerability profile, alongside a low potential for abuse, has also been confirmed in naturalistic settings [5,9,10,11].

It is noteworthy that depression can have a heterogeneous presentation with symptoms embracing emotional, physical, and cognitive domains [12], and that specific clinical phenotypes may affect patients’ response to glutamatergic agents [13]. The effectiveness of treatments is mainly based on objective criteria and clinicians’ evaluation; however, including patients’ self-rated, lived experience on changes in their symptomatology might be useful to understand the perceived helpfulness of therapies and to fill the knowledge gap about treatment outcomes [14,15]. Preliminary data focusing on clinician-rated specific symptoms and psychopathological dimensions reported beneficial effects of ESK-NS on anxiety, cognition, and physical correlates, as well as on anhedonia and suicidality in TRD patients [2,16,17,18]. However, little evidence is available on the viewpoint of patients with TRD receiving esketamine, and it has been investigated through audio interviews [19] and self-administered questionnaires assessing cognition, quality of life, and functional outcomes [18,20,21].

Therefore, this report aims to describe the effects of three-month treatment with ESK-NS in patients with TRD on core symptoms of depression (i.e., mood, anhedonia) and correlated symptomatology (i.e., sleep, cognitive functioning, suicidality, anxiety) considering the patients’ perspective alongside the clinicians’ evaluation.

## 2. Materials and Methods

### 2.1. Participants

Patients referring to the Psychiatric Unit of the Fondazione Policlinico Universitario Agostino Gemelli IRCCS were consecutively included. Eligibility for inclusion was established as follows: a primary diagnosis of major depressive disorder (MDD) according to DSM-5-TR criteria [22]; experiencing a major depressive episode (MDE) under current antidepressant therapy with selective serotonin reuptake inhibitors and/or serotonin/norepinephrine reuptake inhibitors; insufficient response to two or more antidepressant treatments from at least two different classes, assumed at an adequate dosage for 6 to 8 weeks, during the present episode (TRD) [23]. Patients were excluded if they had current medical or psychiatric contraindications to esketamine treatment (e.g., pregnancy, cardiovascular diseases, substance and/or alcohol use, psychotic symptoms, neurocognitive disorders).

### 2.2. Procedures

Esketamine nasal spray was administered in addition to ongoing oral antidepressants, for approved therapeutic indications, twice a week for the first month and, subsequently, once a week over the following 8 weeks, for an overall treatment of three months duration. The first ESK-NS administration consisted of either 28 or 56 mg according to a cut-off age of 65 years and subsequent assumptions were increased to 56 or 84 mg based on clinical judgement and tolerability. Concomitant medications were allowed (see Section 3) and were not modified throughout the observation period.

Sociodemographic (age, gender, educational level, occupation, marital status) and clinical data (age of MDD onset and lifetime number of MDEs, duration of illness, suicidality—both ideation and attempts, hospitalizations, family history of psychiatric disorders, psychiatric and medical comorbidities, smoking) were collected. Comorbid psychiatric diseases and personality disorders were evaluated by trained clinicians using, respectively, the Structured Clinical Interview for DSM-5 Disorders, Clinician Version (SCID-5-CV) [24] and the Structured Clinical Interview for DSM-5 Personality Disorders (SCID-5-PD) [25]. Patients underwent a preliminary evaluation through the Mini-Mental State Examination (MMSE), and the presence of neurocognitive disorders was excluded for MMSE total scores >26 [26].

A psychometric assessment with clinician-rated scales and patients’ self-reported questionnaires was carried out at baseline and after 12 weeks of treatment, including intermediate evaluations at 4 and 8 weeks, to investigate depressive symptoms and psychopathological dimensions. The assessment was performed in the outpatient setting before ESK-NS administrations using the Italian-validated versions of the psychometric tools.

Specifically, depressive symptoms severity was investigated through the total scores of the Montgomery-Åsberg Depression Rating Scale (MADRS) [27] and the Beck Depression Inventory (BDI) [28]. Patients were considered remitters if they obtained a total MADRS score <10 and responders for ≥50% reduction in baseline scores at any time-point (one month, two or three months).

Regarding other psychopathological dimensions of interest, the presence of (i) anhedonia, (ii) sleep alterations, (iii) cognitive symptoms, (iv) suicidality, and (v) anxiety was evaluated through: (i) subitem 8 of MADRS and the total score of Snaith-Hamilton Pleasure Scale (SHAPS) [29]; (ii) subitem 4 of MADRS and subitem 16 of BDI; (iii) subitem 6 of MADRS, Digit Symbol Substitution Test (DSST) [30] and Perceived Deficits Questionnaire for Depression—5 items (PDQ-D5) [31]; (iv) subitem 10 of MADRS and subitem 9 of BDI; and (v) the total scores of the Hamilton Anxiety Rating Scale (HARS) [32] and of the Self-Rating Anxiety Scale (SAS) [33].

### 2.3. Data Analysis

Descriptive data were summarized as the number of patients and percentage (%) or mean ± standard deviation (M ± SD) for categorical and continuous variables, respectively. After controlling for the parametric/non-parametric distribution of variables, the relationship between clinician-rated and patients’ self-report instruments was investigated through a Pearson correlation. The outcome measures—the mean changes from baseline to 1, 2, and 3 months of each efficacy variable—were analyzed using a mixed model for repeated measurements (MMRM), including time as a fixed effect, the baseline score as a continuous covariate, and the baseline score-by-time interaction, based on all available observations. Analyses were performed on all patients with at least one valid post-baseline assessment of the variables (full-analysis set, FAS). A significance level of *p* < 0.05 was used. All analyses were performed using IBM SPSS Statistics for Windows, v. 28 (IBM Corp., Armonk, NY, USA).

## 3. Results

Sociodemographic and clinical characteristics are summarized in Table 1.

At baseline, all patients displayed moderate to severe depression according to MADRS and BDI scores together with clinically significant psychopathology, and overall improved throughout treatment with a mean ESK-NS dose of 78.4 ± 11.43 mg. A significant, positive correlation was found between the following clinician- and patient-rated psychometric tests assessing, respectively, core symptoms of depression (mood and anhedonia) and correlated symptomatology (sleep alterations, cognitive symptoms, suicidality, and anxiety): MADRS—BDI total scores, Pearson’s r = 0.65 (*p* < 0.001); MADRS item 8—SHAPS total score, Pearson’s r = 0.47 (*p* < 0.001); MADRS item 4—BDI item 16, Pearson’s r = 0.34 (*p* = 0.01); MADRS item 6—PDQ-D5 total score, Pearson’s r = 0.59 (*p* < 0.001); MADRS item 10—BDI item 9, Pearson’s r = 0.57 (*p* < 0.001); and HARS—SAS total scores, Pearson’s r = 0.86 (*p* < 0.001).

Clinicians’ evaluation reported significant reductions in MADRS total score and subitems 8 (anhedonia), 4 (sleep), and 10 (suicide), as well as for HARS (anxiety) starting in the first month of treatment, while cognition improved from the second month as for MADRS subitem 6 and the performance at DSST cognitive task. Patients rated early reductions in BDI total scores and subitem 16 (sleep) and SAS (anxiety) after one month, and significant improvements of SHAPS (anhedonia) and PDQ-D5 (cognition) at the second month of treatment, while BDI subitem 9 (suicide ideation) reduction started at one month and became statistically significant at endpoint (Table 2).

Six patients prematurely discontinued treatment with ESK-NS at different follow-ups: three patients withdrew after, respectively, the first administration, one month, and two months of treatment because of clinical reasons related to their medical comorbidities; two patients discontinued ESK-NS during the third month, before reaching the endpoint, due to lack of perceived benefit; and one discontinued because of a relapse into substance use. For patients with all available observations (*n* = 19), response and remission were detected in 52.6% and 31.6% of cases at different time-points (*n* = 4 responders, *n* = 1 remitter at two months; *n* = 6 responders, *n* = 4 remitters at three months). One patient achieved remission after the first month of treatment.

None of the participants experienced clinically relevant side effects throughout the treatment period. Patients developed mild nausea and small increases in blood pressure during the two-hour post-administration, generally more intense for the first 40 minutes depending on the peak blood level of esketamine, which were well tolerated and resolved over post-administration observation. Mild and transitory dissociation was mainly detected within the 40 minutes peak after the first administrations (as for total scores > 4 at the Clinician Administered Dissociative State Scale, CADSS) and progressively reduced under the threshold value over subsequent sessions [34].

## 4. Discussion

To our knowledge, this is the first report describing the effects of esketamine nasal spray from the patients’ perspective on core symptoms of treatment-resistant depression and correlated psychopathological dimensions. Patients from this sample presented moderate to severe depression before undergoing ESK-NS and improved throughout treatment.

A 50% or greater reduction in depressive symptoms is usually considered a response to antidepressants in clinical trials [35]. Our results showed similar rates at the endpoint in a population with relevant clinical severity, except for one patient who rapidly reached remission. The possibility of a delayed response in cases with higher degrees of treatment resistance has been postulated in relation to esketamine’s effectiveness [36]. Indeed, recent studies outlined a greater response at three months in real-world samples supporting the long-term efficacy of ESK-NS continuation in patients with TRD who do not achieve full remission after the first month [11].

The number of MDEs that occurred in a lifetime, the current MDE lasting more than one year, the presence of suicide ideation in almost half of cases, significant anxiety, as well as organic and psychiatric comorbidities support the clinical severity and the subsequent burden of this TRD sample. The relapsing–remitting course of MDD can involve a mean of up to four depressive episodes per patient, with recurrences displaying increased severity, more chronicity, and a lower probability of response [12]. Specifically, recurrent depression has been associated with higher rates and intensity of depressive symptoms, pervasive pessimistic and suicidal thoughts, somatic disturbances, and cognitive dysfunction, with severity increasing alongside the number of episodes [12,37]. The presence of comorbid anxiety has also been demonstrated to combine with higher chronicity, more suicide attempts, and reduced response–remission rates to antidepressants during a depressive episode [38], thus being a predisposing factor to TRD [39,40]. Additionally, the use of benzodiazepine compounds (as for almost half of the sample in this study) has been recently listed among predictors of delayed response to ESK-NS, arguing that the former could slow esketamine action due to the contrasting effects on the glutamatergic system [2]. However, no significant interactions or negative impact on the effectiveness of ESK-NS had been previously reported for the concomitant assumption of sedative-hypnotics/anxiolytics [41], and growing evidence shows ketamine and esketamine’s anxiolytic effects in both unipolar and bipolar TRD, with a positive impact of anxious symptoms on response to ESK-NS [2]. Here, we detected significant levels of anxiety at baseline and their rapid improvement seems in line with most recent findings.

TRD patients also presented psychiatric and medical comorbidities, which were under maintenance pharmacological treatment (i.e., in remission and/or without significant alterations of monitoring parameters) and did not fall within ESK-NS contraindications. Co-occurring psychiatric diseases and general medical conditions can be involved in treatment resistance and should be addressed to reduce the overall burden of symptoms [42]. The intake of polypharmacotherapy is usually required to handle complex clinical presentations, becoming an exclusion criterion for most randomized clinical trials [43], and can further influence treatment-response because of drug interaction, as it has been reported for ketamine, mood stabilizers, and antipsychotics, which are the compounds most frequently used in augmentation strategies [3,44].

Within this background, we may assume that specific clinical pictures of patients might have influenced, in this study, the symptomatic course during treatment with ESK-NS. Clinicians rated early improvements (i.e., from the first month) in nearly every symptomatic dimension, and the patients’ perceptions were consistent in most of the symptoms (i.e., mood, sleep, anxiety, and cognition). The substantial overlap of clinicians’ and patients’ evaluations could be explained by considering the correlations detected in psychometric assessments administered to this sample, in line with recent results from a factor analytic study that compared self-report and observer-rated scales in depression [45].

It is known that quantifying depression can vary depending on the questionnaires chosen [46]. Several reasons contributing to the discrepancy between self- and clinician-rated scales have been reported, such as the bias of depression severity and the inability to adequately assess some psychopathological dimensions for self-ratings as well as expectations of the clinicians about treatments, especially within naturalistic studies [45,46]. Here, a delayed improvement was reported in anhedonia and suicide after the second and third month of treatment, respectively.

Anhedonia, defined as “markedly diminished interest/pleasure in all/almost all activities [22]”, is a core symptom of depression and stands out as a key target of treatments, including glutamatergic antagonists [47,48]. The original concept of anhedonia has been expanded over the last decades to include a spectrum of reward-processing deficits, which underlie several psychiatric disorders beyond MDD and may explain the anti-anhedonic effect of ketamine and its derivatives [49]. Dysfunctions in fronto-striatal dopaminergic circuits are the main neurophysiological basis of anhedonia [50] and the role of prefrontal areas in modulating reward mechanisms has been demonstrated to involve glutamatergic pathways [51]. Reverting the glutamatergic hypoactivation in these networks could restore an appropriate hedonic function, as reported after ketamine and esketamine administrations in both unipolar and bipolar depression [52]. Preliminary evidence pointed to positive effects on anhedonia also for the intranasal route and, vice versa, the presence of anhedonia has been identified among predictors of positive outcomes (both response and remission) after three months of treatment with ESK-NS [2].

Suicide has become a major public health concern, especially in relation to depression, with approximately 30% of subjects affected by depressive disorders who attempt suicide every year [53]. The anti-suicidal properties of glutamatergic antagonists have been reported and systematic reviews with meta-analysis highlighted positive outcomes for intravenous ketamine and ESK-NS vs. placebo in patients with TRD and suicidality [16,54,55]. Conversely, recent evidence identified high baseline suicidality as a predictor of poor response to ESK-NS at one and three months of treatment [2].

In this sample, patients showed a delayed perception of the improvement in anhedonia and suicidality and some considerations can be made in support. According to baseline measures, 76% of patients achieved and, in most cases, far exceeded the cut-off score for anhedonia (SHAPS > 2), while suicide had been a concern in 68% of the sample. Evidence from the literature has described the link between higher levels of anhedonia with greater depression severity and, specifically, with more lifetime depressive episodes, longer episode duration, higher recurrence, and persistence of the disease [49]. Further, anhedonia has been identified as a significant risk factor for suicide in general and psychiatric populations [56], even independently of depression severity [49].

Growing evidence highlighted how different symptomatic clusters can characterize major depression and their potential association with specific neurobiological mechanisms, including inflammatory alterations [57]. The role of inflammation has been increasingly observed in several mental illnesses and subgroups of psychiatric symptoms across diagnoses [58]. The efficacy of anti-inflammatory treatments in reducing anhedonia seems to support the involvement of inflammation in the pathophysiology of such dimension [59,60]. Also, suicide pathogenesis has been associated with neuroinflammation [61] with a specific interrelationship that goes beyond the underlying psychiatric illness, such as major depression [62]. However, the brain-derived neurotrophic factor (BDNF) has been reported among the underlying mechanisms of the association between suicidality and depression [63]. Besides the inhibition of NMDA receptors, ketamine and esketamine can exert favorable effects on neuroinflammation through the modulation of BDNF and other intracellular proteins like the mechanistic target of rapamycin complex 1 (mTORC1) [18]. Indeed, an anti-inflammatory effect has been reported for both compounds with a potential enhancement of their anti-depressive properties [64].

Therefore, although further studies with larger samples are needed, we could argue that patients with TRD and concomitant high levels of anhedonia and suicidality, like those from this sample, might need more time to feel a clear enhancement in certain symptomatic dimensions, according to the greater severity that characterizes TRD [65], the inflammatory theory arisen in the field of mood disorders, and delayed onset of statistically significant improvements in ESK-NS-treated patients with more complex, chronic forms of depression with specific prognostic factors [2,66].

Since the presence of anhedonia tends to negatively affect treatment initiation, engagement, and compliance with poor treatment outcomes, and considering the interconnection with suicide, psychotherapies focused on increasing the perceived value of treatment during initial sessions (e.g., by emphasizing positive feedback and treatment benefits in the short term through social and financial initiatives) [67] could improve both conditions and, thus, should be considered in addition to psychopharmacological interventions to implement effectiveness and outcomes of treatment [56]. Moreover, given the symptomatic heterogeneity of depressive disorders, identifying the clinical specificity profiles of patients and including their own perspective on mental health and wellbeing as “experts by experience” [15] becomes fundamental in personalized medicine to deliver targeted approaches.

A first step towards precision psychiatry has been made through the development of novel rapid-acting antidepressant drugs, and recent evidence has been collected about their effect on different symptom domains [68]. However, more challenging cases of TRD may need multidisciplinary interventions that could comprise self-help settings, psychotherapeutic approaches, functional rehabilitation, and promotion of neuronal plasticity, beyond pharmacological interventions [42], to improve the patient’s functioning and quality of life alongside symptom reduction [69].

Some limitations should be outlined, like the small sample size and lack of a control group and specific, standardized tools for patients’ rating of TRD (e.g., Massachusetts General Hospital—Antidepressant Treatment Response Questionnaire, MGH-ATRQ) in the psychometric assessment. Also, the specificity of the sample with a relevant clinical severity, which could have influenced the symptomatic course and treatment response, and the monocentric setting might reduce the generalizability of the results. Nevertheless, this study has some strengths, like the emphasis on patients’ perspective in the interpretation of treatment outcomes, a longitudinal design with a three-month follow-up, and the use of validated, standardized tools (including a specific task for cognitive functioning) to assess different symptomatic/psychopathological dimensions.

## 5. Conclusions

The increasing prevalence and the clinical and social implications of TRD, which are associated with an overall high clinical severity and detrimental outcomes, point to the need for a better characterization of the clinical phenomenology of patients to deliver personalized interventions, improve long-term outcomes and enable a full functional recovery. It has been demonstrated that self-reports and clinician ratings should be considered complementary and equally useful to measure multiple aspects of depression [45]. Moreover, given the association between specific symptomatic dimensions and neurobiological mechanisms [57], the inclusion of biomarkers and a correlation of biological variables with psychometric scores could improve the understanding of substrates [12] as well as the identification of more reliable outcome predictors in difficult-to-treat populations like patients with TRD. Therefore, the present study supports the utility of a careful clinical and psychometric assessment that encompasses both clinicians’ and patients’ perspectives and suggests specifically targeting prognostic factors like anhedonia and suicidality [56,70,71]. A synergistic approach during treatment should enhance esketamine’s efficacy and accelerate improvement, and its subjective perception, also in patients with symptomatic profiles that might hinder a prompt response. These findings pave the way for future, larger, real-world trials specifically aimed at defining the symptomatic profiles of patients and how these might influence treatment response.

## Figures and Tables

**Table 1:** Sociodemographic and clinical characteristics at baseline.

	N (%); M ± SD
**Overall**	25
*Sociodemographic features*	
**Age** (years)	55.1 ± 10.9
**Gender**	
Female	11 (44)
Male	14 (56)
**Education level** (years)	13.8 ± 3.73
**Occupation** (employed)	9 (36)
**Marital status** (married)	11 (44)
*Clinical data*	
**Age of onset** (years)	30.3 ± 11.6
**Lifetime MDEs** (number)	3.48 ± 2.35
**Duration of current MDE** (months)	18.8 ± 11.3
**Suicidality**	
Ideation	13 (48)
Attempts	5 (20)
**Psychiatric hospitalizations**	11 (44)
**Antidepressants**	
SSRIs	17 (68)
SNRIs	9 (36)
Others	10 (40)
**Other psychopharmacotherapy**	
Mood stabilizers/Anticonvulsants	14 (56)
Antipsychotics	11 (44)
Sedative hypnotics/anxiolytics	12 (48)
**Psychiatric comorbidities**	12 (48)
Substance use disorders	4 (16)
Eating disorders	2 (8)
Personality disorders	3 (12)
Anxiety disorders	2 (8)
Obsessive compulsive disorders	1 (4)
**Family history of psychiatric diseases**	21 (84)
**Medical comorbidities**	18 (72)
Essential hypertension	7 (28)
Gastrointestinal diseases	5 (20)
Respiratory diseases	3 (12)
Osteoarticular diseases	2 (8)
Metabolic diseases	2 (8)
**Smoking habits**	8 (32)
**MMSE**	29 ± 1.84

Abbreviations: M, Mean; MDE, Major Depressive Episodes; MMSE, Mini-Mental State Examination; SD, Standard Deviation; SSRIs, Selective Serotonin Reuptake Inhibitors; SNRIs; Serotonin Norepinephrine Reuptake Inhibitors.

**Table 2:** Psychometric features at baseline and effect of ESK-NS on depression and other psychopathological dimensions (FAS, MMRM).

		Mean Change from Baseline (SE)					
**Psychometric assessment** (M ± SD)	Baseline	1 month	*p*	2 months	*p*	3 months	*p*
*Clinicians’ perspective*							
**MADRS**	34.2 ± 8.15	−7.37 (1.90)	**<0.001**	−7.10 (1.71)	**<0.001**	−8.67 (1.99)	**<0.001**
Sleep	3 ± 1.41	−1.45 (0.33)	**<0.001**	−1.01 (0.34)	**0.004**	−0.71 (0.35)	**0.04**
Cognition	3.64 ± 1.15	−0.33 (0.32)	0.293	−0.95 (0.32)	**0.005**	−1.17 (0.33)	**<0.001**
Anhedonia	4.20 ± 1.04	−0.83 (0.28)	**0.005**	−1.09 (0.29)	**<0.001**	−1.42 (0.30)	**<0.001**
Suicide	2.4 ± 1.58	−0.85 (0.29)	**0.007**	−0.41 (0.30)	0.178	−0.85 (0.32)	**0.01**
**HARS**	20.5 ± 8.51	−5.34 (1.60)	**0.002**	−4.61 (1.47)	**0.003**	−4.55 (1.61)	**0.007**
**DSST**	39.1 ± 13.2	2.29 (1.78)	0.205	5.13 (1.66)	**0.004**	4.86 (1.74)	**0.008**
*Patients’ perspective*							
**BDI**	30.9 ± 10.8	−5.67 (2.61)	**0.036**	−6.08 (2.26)	**0.01**	−8.71 (2.71)	**0.003**
Sleep	1.47 ± 0.841	−0.46 (0.21)	**0.036**	−0.46 (0.22)	**0.038**	−0.15 (0.23)	0.525
Suicide	1.42 ± 0.902	−0.36 (0.20)	0.076	−0.3 (0.20)	0.48	−0.45 (0.21)	**0.039**
**SHAPS**	6.85 ± 3.62	−1.04 (0.77)	0.181	−1.38 (0.68)	**0.04**	−2.38 (0.82)	**0.006**
**SAS**	47.5 ± 8.91	−4.68 (1.81)	**0.015**	−4.16 (1.70)	**0.02**	−4.72 (1.95)	**0.02**
**PDQ-D5**	9.45 ± 4.89	−1.50 (0.82)	0.072	−2.19 (0.71)	**0.003**	−2.15 (0.87)	**0.02**

Significant results in **bold**. Abbreviations: BDI, Beck Depression Inventory; DSST, Digit Symbol Substitution Test; ESK-NS, Esketamine Nasal Spray; FAS, Full Analysis Set; HARS, Hamilton Anxiety Rating Scale; M, Mean; MADRS, Montgomery–Åsberg Depression Rating Scale; MMRM, Mixed Model for Repeated Measures; *p*, statistical significance; PDQ-D5, Perceived Deficits Questionnaire for Depression—5 items; SAS, Self-Rating Anxiety Scale; SD, Standard Deviation; SE, Standard Error; SHAPS, Snaith–Hamilton Pleasure Scale.

## Data Availability

Authors do not have permission to share these data.
